# Erie County Medical Society

**Published:** 1846-02-01

**Authors:** 


*Erie County Medical Society.*—The annual meeting of this Society was held on the 13th ult., at the Lecture Room of the Young Mens' Association. The report of the Committee appointed at the semi-annual meeting to take into consideration the subject of medical legislation and reform, and report thereupon for the action of the Society, in conformity with the request of the State Society, occasioned considerable discussion. The majority of the Committee reported two resolutions—one recommending that no application be made to the Legislature for new laws or for the alteration of existing laws, but if it be deemed expedient to make any application, it should be to abolish all laws regulating the practice of physic and surgery. A minority of the Committee were in favor of changing the present mode of conferring licenses and diplomas, depriving Medical Colleges and County Societies of this privilege, and confining it to a single, central board of examiners, or to a board for every senatorial district. The Society sustained the resolution reported by the majority of the Committee. The Committee, also, reported a resolution approving of the plan of a National Convention, which was adopted by the Society.

The annual address was delivered by Dr. John S. Trowbridge. The subject was Puerperal Convulsions. It was a creditable performance. The Doctor impressed the importance of being prepared for this truly terrible complication of the puerperal state by sound views of its pathology, and correct principles of treatment. Fortunately the disease is rare, but every practitioner is daily liable to meet with it, under circumstances which will be most trying to his feelings, and which will call for the immediate application of all his knowledge, and his best judgment. The distinctions between it and Epilepsy, and Hysteria, were pointed out. Dr. S. regards it as a peculiar affection of the nervous system, with a tendency to conditions of the nervous centre analogous to those which obtain in Apoplexy. The importance of recognizing premonitory indications of its development, and prompt resort to an active depletory treatment in all cases where the diagnosis is certain, or even doubtful, was duly considered.

The address of the President, Dr. Wakeley, on retiring from office, was postponed until the next semi-annual meeting.

Dr. Wilcox, of this city, was appointed to deliver the address at the next meeting of the Society.

The following are the officers elected for the present year:

President, F. L. Harris, M. D.; Vice President, Isaac Parsells, M. D.; Secretary, Charles Winne, M. D.; Treasurer, H. N. Loomis, M. D.; Librarian, Josiah Trowbridge, M. D.; Censors, Drs. Scott, Pride, G. N. Burwell, J. S. Trowbridge, and Mixer.

